# Learning Assurance Analysis for Further Certification Process of Machine Learning Techniques: Case-Study Air Traffic Conflict Detection Predictor

**DOI:** 10.3390/s22197680

**Published:** 2022-10-10

**Authors:** Javier A. Pérez-Castán, Luis Pérez Sanz, Marta Fernández-Castellano, Tomislav Radišić, Kristina Samardžić, Ivan Tukarić

**Affiliations:** 1 ETSI Aeronáutica y del Espacio, Plaza Cardenal Cisneros, Universidad Politécnica de Madrid, 28008 Madrid, Spain; 2 Faculty of Transport and Traffic Sciences, University of Zagreb, Borongajska Cesta, 10000 Zagreb, Croatia

**Keywords:** air transport, conflict detection, machine learning, learning assurance, trustworthiness

## Abstract

Designing and developing artificial intelligence (AI)-based systems that can be trusted justifiably is one of the main issues aviation must face in the coming years. European Union Aviation Safety Agency (EASA) has developed a user guide that could be potentially transformed as means of compliance for future AI-based regulation. Designers and developers must understand how the learning assurance process of any machine learning (ML) model impacts trust. ML is a narrow branch of AI that uses statistical models to perform predictions. This work deals with the learning assurance process for ML-based systems in the field of air traffic control. A conflict detection tool has been developed to identify separation infringements among aircraft pairs, and the ML algorithm used for classification and regression was extreme gradient boosting. This paper analyses the validity and adaptability of EASA W-shaped methodology for ML-based systems. The results have identified the lack of the EASA W-shaped methodology in time-dependent analysis, by showing how time can impact ML algorithms designed in the case where no time requirements are considered. Another meaningful conclusion is, for systems that depend highly on when the prediction is made, classification and regression metrics cannot be one-size-fits-all because they vary over time.

## 1. Introduction & Literature Review

Air traffic has suffered the largest collapse in its history due to COVID-19. Lockdown across the world implied that the top movements for 2019 dropped by around 80% in 2021 [1]. However, air traffic is recovering rapidly, and the aviation industry must face the same problems as in 2019. The Single European Sky’s ATM Research Project (SESAR) informed in the Air Traffic Management (ATM) master plan capacity problems [2]. Due to the increase in air traffic expected for 2035 and 2050, it must be handled by the introduction of automation and new technologies based on Artificial Intelligence (AI).

European Union Aviation Safety Agency (EASA) defines AI as ‘any technology that appears to emulate the performance of a human’ [3]. One of the branches of AI is data-driven approach (statistical AI) and Machine Learning (ML) is one of the branches that takes advantage of historical data. The European Aviation AI high-level group defines ML as ‘the ability of algorithms to learn from the input and output data that characterise them’ [4]. ML algorithms aim to learn patterns from databases (historical situations that have been previously laid out). The output of the ML algorithm is the ability to apply learned rules or algorithms to predict new situations. ML algorithms can be applied to classification or regression techniques. Classification algorithms perform predictions based on discrete output variables (they can be binary or multiclassification problems), and regression algorithms perform numerical predictions. The authors recommend the previous work of [5] to deepen these topics.

One of the main hazards of using ML systems is to avoid the ‘black box’ effect. Main concerns about AI-based systems are related to learning assurance, explainability, and trustworthiness. Several works are dealing with these issues, bringing to the light novel solutions [6,7,8]. EASA published the design assurance concepts for neural networks that apply also for ML techniques [9]. The main goal of this work is to develop a learning assurance process that enriches trustworthiness of AI-based systems. The main result was the identification of a W-shaped development process for ML applications. This W-shaped has been considered critical for the further development of AI-based systems to ensure the learning process. EASA developed its first usable guide for ML application [10]. It develops the process to be followed to cover some aspects related to learning assurance, explainability and trustworthiness, and is one of the pillars for the further certification process for AI-based systems. 

Aviation is a field extremely prone to the use of AI-based techniques because of the huge amount of data that can be available. ML techniques have recently been applied to different topics: trajectory prediction [11,12,13], airspace performance metrics [14,15] and atmospheric models [7,16]. Herein, ML techniques are applied to Conflict Detection (CD) in Air Traffic Control (ATC). CD is based on a tactical level where it has to provide information to the ATC Officers (ATCOs) about separation provision. Therefore, CD is designed as a tool to help ATCOs identify conflicts in airspace. CD is not novel and has been studied during the last decades with different approaches. Krozel et al. [17] proposed a division into four areas depending on the mathematical model used: static, dynamic, uncertainty, and probability. Paielli et al. [18] developed a new approach based on tactical pairwise trajectory analysis. Other authors found their studies on complex probabilistic models to understand trajectory uncertainty [19,20,21]. 

The novelty of this work does not fall in the CD model based on ML techniques because the concept has already been described in [22]. Therefore, the main contributions of this paper to the literature are as follows:

- This is one of the first learning assurance analyses performed for CD predictors in ATC.
- The results are crucial to understand that time dependence must be considered in most of the AI-based systems.
- The W-shaped methodology needs to refine the procedure before being considered for certification issues.
- One-size-fits-all statistical measures for ML algorithms cannot be accepted without an in-depth analysis.

The rest of the paper is structured as follows. Section 2 provides insights of the conflict detection operational concept; the trajectories source and they have been treated to constitute the database. Section 3 describes the learning assurance process followed for the ML techniques. Section 4 shows the results obtained from the time dependence analysis of the CD tool, analysing regressive and classification predictions. Lastly, conclusions and further work are summarised in Section 5. 

## 2. Conflict Detection Concepts

**Figure 1:**
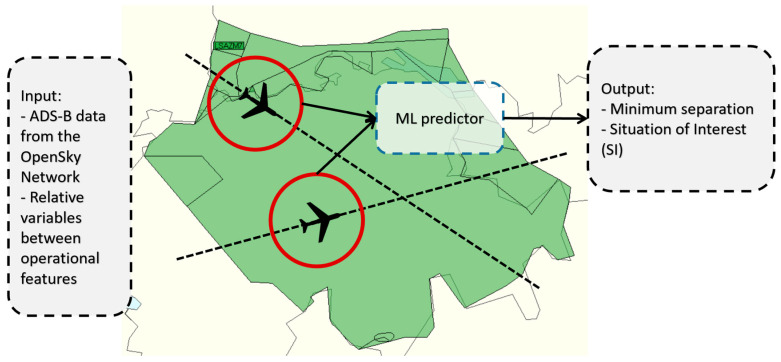
Operational concept of the CD tool.

This tool aims to perform CD predictions among pairs of aircrafts in the airspace. Conflict is defined by ICAO as any situation where the separation minima could be a compromise in the near future [23]. One distinguishing feature of the CD tool is that it does not perform trajectory predictions and then analyses separation infringements, but it performs predictions for separation infringements based on historical data. This section is a summary of the work previously developed by the authors in [22,24]. Figure 1 shows a summary of the operational capability of the CD tool. 

Therefore, the basics of the CD tool are simple because it demands some input values to feed the ML predictor and some outputs are obtained. The database to train the ML algorithm is based on ADS-B data from the OpenSky Network [25] and the main variables used are:

1. Position: Longitude (*λ*), latitude (*ϕ*) and altitude (*h*).
2. Velocity: Ground speed (*GS*) and vertical rate ($\underset{h}{˙}$).
3. Heading (*θ*).
4. Relative variables based on previous variables.
5. Target variables based on 4DT predictions.

The total number of variables used is 36, although there are many that do not provide information for the ML algorithm and are removed during the featuring engineering. Labels or targets (variables to predict) are:

6. Minimum distance (*MinDis*): A numerical variable of the minimum distance expected to reach between an aircraft pair.
7. Situation of interest (*SI*): Binary variable that classifies aircraft pairs as *SI* or *No SI*.

Current separation minima in en-route airspace are longitudinally 5 Nautical Miles (NM) and vertically 1000 feet (ft). When dealing with ML classification techniques, the main problem is located on the border. Avoid this problem with the current separation minima, the concept of situation of interest (SI) has been considered. One SI is a situation in which an aircraft pair is expected to intersect with a horizontal separation smaller than a predefined distance. In this work, an SI was defined as 10 NM and 1000 ft. Typically, this separation is specified in advance by Air Navigation System Provider (ANSP) and would be studied in further work.

In addition, it is important to note that the CD tool is designed in order not to present time dependence for predictions. In other words, the predictions are independent because the prediction of previous timestamps is not used to become a further one. Therefore, each sample (each timestamp for every aircraft pair) represents a different situation that is independent of the rest of the situations in the database.

This work selected the LSAZM567 airspace sector of the Zurich airspace with vertical boundaries from Flight Level (FL) 355 to FL660. It focuses on en-route airspace characterised by a low rate of climbing and descending aircraft and high number of cruise operations. The time period considered is the first 15 days from the AIRAC cycle of June 2019.

## 3. Material & Methods: Learning Assurance Process for AI-Based Systems

EASA has proposed a new W-shaped methodology to provide a first roadmap of direction for AI-based projects that could help in level 1 ‘human assistance’ [10]. This guide document provides crucial information for the future means of compliance of AI-based systems and the way the development process should be. Every AI-based system should deal with the following analyses:

1. Learning assurance: ensures the technical development to cover learning processes specific for AI- based systems
2. Explainability: refers to the ability to provide relevant and understandable information on how the AI application is coming to its results.
3. Safety risk mitigation: performs a safety risk analysis and mitigation process in order not to be able to open the AI black box.

Learning assurance analysis allows to dig into the development process of the AI-based system and the primary analysis of its performance. Explainability is the second step, but its analysis and results strongly depend on the relation between the system and the human: what information s/he needs, when and why. Lastly, safety risk mitigation must ensure a correct analysis and mitigation process about the introduction of the AI-based system before an operational phase. Therefore, this work is narrowed to learning assurance and further work could analyse the implementation of CD in a real environment.

**Figure 2:**
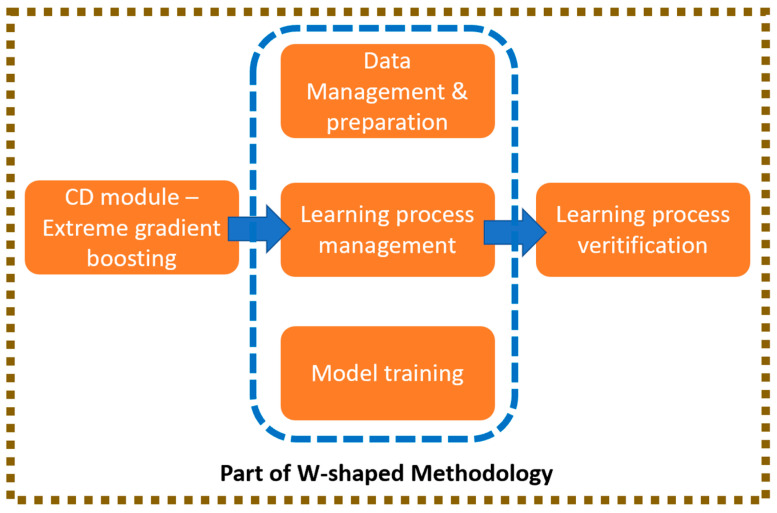
Specific areas of W-shaped methodology evaluated.

The W-shaped methodology is substantiated by 11 sequential steps, beginning from the system requirements & design and finalising it by the verification of these requirements. Figure 2 shows the specific areas of W-shaped methodology evaluated in following subsections.

However, the learning process for a technology readiness level (TRL) 1 [1], as it is the development of this conceptual tool, starts directly on the data management (step 3) and ends on the learning process verification (step 6), covering learning process management (step 4) and model training (step 5). The implementation, integration, and verification of the rolling out process are out of the scope. 

### 3.1. Data Management 

Data management is the first step of the data life-cycle management and covers: data collection, preparation, data set split, validation, and verification. The goal is to ensure the accuracy, validity and integrity of the dataset. It is achieved by the definition of the data requirements related to accuracy, integrity, availability or traceability of the sources. In addition, data collection must ensure that no problems with privacy or personal data can be affected, as well as the identification of the different sources that are relevant for the system. There is an issue that should be studied in the future about the necessity of validating the training data. It can be analysed by statistical methods for generalized performance, but it is not possible to review manually millions or thousands of samples.

In this work, there are no specific data requirements, and the trajectories are obtained from the OpenSky Network [25]. Currently the OpenSky Network is the only available (under license) source of ADS-B data. It can be not the most accurate data source, but it meets the requirements of the concept because it allows to have access to thousands of trajectories. Further work could take advantage of other trajectories provided by ANSPs or airlines depending on the necessities or the requirements. 

### 3.2. Data Preparation

Data preparation is a multistep process, which is paramount as AI-based systems need the data to learn the underlying relations and develop a mathematical model to make predictions. The main areas are as follows:

1. Data pre-processing: It is the first step and consists of preparing the data for the feature engineering or the learning process. The trajectories obtained from the OpenSky Network required the following preprocessing:
  ○ Duplicated trajectories are removed.
  ○ Trajectories with at least 10 ADS-B erroneous data are removed.
  ○ Trajectories that do not belong to the airspace boundaries of LSAZM567 are removed.
  ○ The NaN or missed data are filled with the average value based on the previous and subsequent ADS-B data.
  ○ Aircraft pairs are generated by clustering by day and hour.
  ○ Trajectories are temporarily modified to penetrate the airspace at a similar time span to simulate separation infringements.

The main issues during the preprocessing were the computational time and workload required to handle the large number of samples, lack of separation infringements although aircraft were temporarily modified, and weather could not be possible to include it.

2. Feature engineering: consists of transforming the preprocesed data to improve the representativeness of the underlying structure. Here, geospatial latitudes and longitudes were separated into different variables, new variables based on relative operational variables are added for each aircraft pair, categorical variables were modified following a one-hot encoding process, and timestamp variables were removed.

3. Normalisation: rescales the values of numeric columns without creating differences in the ranges of values. There are several methods available for normalization: zscore was selected because it normalizes the numerical values of each variable by fitting to a normal distribution.

4. Data allocation: is the last step and splits the database into three datasets:
  ○ Training, validation and testing set. Training set (63% of the whole database to train the model), validation set (27% of the entire database to optimise the model) and testing set (10% of the whole database to analyse the final performance of the model).
  ○ Shuffling: It distributes the samples randomly into the training and validation datasets.
  ○ Stratification: Apart from randomly distributing the samples, the stratification process spreads the samples keeping the SI statistical distributions.

Therefore, the database is made up of 60,469 pairs of aircraft and 2,841,803 samples. Each sample represents an aircraft pair situation at a specific time stamp. There are aircraft pairs constituted from 1 up to 900 samples depending on time flying until the CPA. Table 1 shows the statistical data extracted from the main variables.

This data must be used for a twofold purpose: first) experts judgement ensures the validity of the dataset statistics, and second) it is used to analyse the validity of the predictions during the learning process verification. 

### 3.3. Learning Process Management

The management of the learning process considers the preparatory step of the formal training phase. It must fulfil the description of AI-based systems architecture, algorithms to be evaluated, model parameters, cost function, hyperparameters, the process to be followed and to quantify the generalisation rate.

The management of the learning process follows the following steps:

1. Analysis of different ML algorithms. The first step is to evaluate the performance of different ML algorithms in order to identify the ML model that provides better results. It has been assessed with 15 ML algorithms for classification and 17 algorithms for regression.

The best algorithms are based on F1 and Root Mean Square Error (RMSE) metrics that provide the most balanced results. F1 is a combination of recall and precision, metrics to measure the accuracy of the predictions based on the false positives and false negatives, respectively, while RMSE is typically the best option because large errors impact the metric more than small deviations. The ML model has been developed in Python^®^. Scikit-learn is the open-source ML library used for Python [2]. PyCaret is another open-source library that simplifies the implementation of ML algorithms [26].

2. Feature selection. The selection of features aims to identify the influence of the different features that make up the database. The feature selection is performed based on graphical analysis and Recursive Feature Elimination (RFE) with cross-validation [26]. RFE analysis evaluates which is the impact of the features on the model accuracy. Finally, the features that do not influence the ML model are removed from the database.
3. Optimisation of the ML algorithm. An optimisation process to improve the selected ML algorithm’s performance is applied based on hyperparameters grid search. Hyperparameters can be defined as the settings of an algorithm that can be adjusted to optimise the model performance. Hyperparameters must be defined in advance of the training process of the model, and it depends on the ML algorithm identified as the best in step 1. In addition, the metric to optimize is different for the classification and regression problems and should be considered in relation to problems related to unbalanced datasets.
4. Finalization of the ML model. Once the ML algorithm is optimised, it is trained on the whole data set (training and validation set). The finished model represents the model that will be implemented in further work and is validated against the testing set.

### 3.4. Training and Learning Process Validation

The training or learning process consists of applying ML algorithms to understand and reproduce the underlying patterns on the training data set. Once the ML model is trained, the model performance is evaluated using the validation data set. Generally, an optimisation process is required to improve the accuracy of the model. 

The main problem during the training and validation process is overfitting (high variance). The bias-variance trade-off should be taken into account in the ML algorithm. The ML algorithm should have a high enough complexity to minimise bias, but not too high to avoid high variance. Regularisation is a typical method to avoid overfitting with complex models such as random forests. This work has taken advantage of cross-validation to avoid overfitting ML models [26]. The training set is divided into k folds, one is considered the validation set, and the rest are regarded as the training set. This process is repeated for every k fold, and the metrics are calculated based on the mean and the standard deviation.

As the database is highly imbalanced (5% of SI samples), cost-sensitive techniques have been applied to deal with this problem [27]. Typically, the cost function of the ML algorithms equally considers the cost of different misclassifying classes. However, the weights for the cost function can be modified in order to increase the impact of misclassified the minority class. Then, the algorithm reduces the misclassification of the minority class by performing a grid search of the correct misclassification weights. 

The ML algorithm developed and used for both experiments was XGboost and the main results after finalising the ML model are shown in Table 2. Specific details of the validation of the training and learning process are described in [24].

In addition, an analysis has been performed that covers the compatibility of the regression and classification predictions. The goal is to use the ability to combine both predictions to inform ATCO only when there is a double prediction match (SI = 1 and MinDis < 10 NM). The combination of both predictions ensures that 99.9% match reality although 16% of the real SI situations are not identified. However, among the real SI situations not identified by the ML algorithm, only 4.5% represents a conflict. Therefore, it can be concluded that the combination of both ML algorithms ensures a 99.9% success rate for SI, reducing the number of missed and false alerts to 0.1%. This is the step at which the CD tool should be confirmed to fulfil the requirements related with prediction ability. 

### 3.5. Learning Process Verification

This is the last step to confirm or disregard the performance of ML modules. Learning process verification consists of the evaluation of the testing dataset with the predictions of ML model already trained. There are four analyses that should be performed in this section:

5. The most important requirement herein is that the testing set should be completely new to ensure a simulated deployment phase. Some variation on the performance could be affordable, but it should not exceed 5–10% to ensure no generalization problems appear. The classification and regression results are bounded by 8% which fulfil this requirement, although it should be improved in further work.
6. A robustness (or stability) analysis of the ML algorithm should be performed. This robustness analysis is normally performed on the basis of sensitivity analysis. The sensitivity analysis is performed varying slightly some values of the operational values to identify if small variations imply large modifications on the ML performance. The fluctuations in the training and testing dataset should be analysed. This analysis was made based on a comparison between the statistical deviation of the predictions compared to the standard deviations. All predictions are covered by 3σ values.
7. Abnormal or adversarial tests are defined for samples with values outside of the limits. In this case, Table 1 has been used as a requirement to define the statistical limits of the operational variables that new samples must meet. In the case there are provided samples that do not match the statistical boundaries, then, it should not be considered.

Therefore, the analysis of the concept developed for this CD system based on ML techniques covers most of the W-shaped methodology. The further development of this system should include the previous steps for the definition of requirements for specific metrics and improving some areas. 

However, EASA does not consider performing expert analysis on how the AI-based system should work. In this work, it seems affordable to assume that the predictions should improve throughout the aircraft pairs are getting closer because the uncertainties should disappear. It could be expected that as the aircrafts approach, the probability of SI (or no SI) will increase and the MinDis prediction should be closer to the real value. In other words, Time-dependency can be crucial in tools that depend on the location of the aircraft and where no specific notation is made.

## 4. Results & Discussion

The CD tool is designed as to be time independent of the predictions. The database has been built considering this concept as explained in Section 3 (timestamp is not a variable on the database). However, it has also been designed to perform predictions in different situations depending on the locations of pairs of aircraft. In other words, there is no direct relation of time-dependency developed on the CD tool, but it should be analysed. 

**Figure 3:**
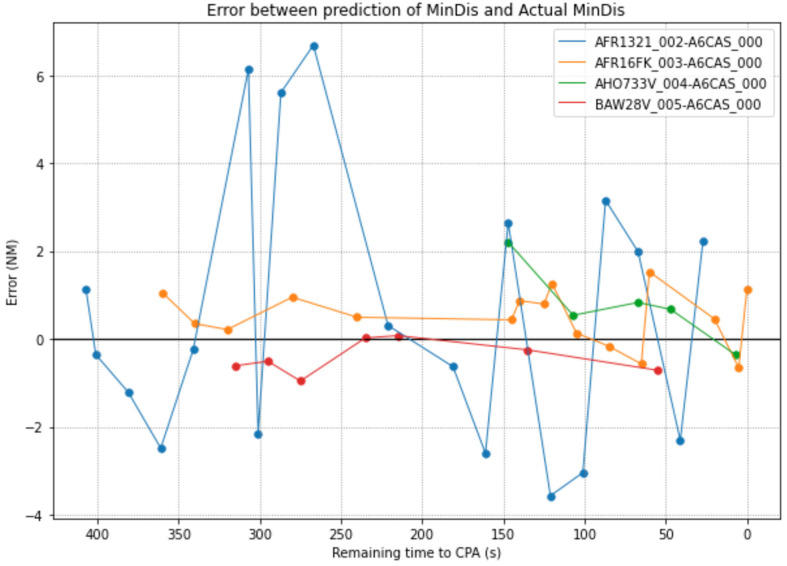
Evolution of 4 aircraft-pairs predictions.

The assumption considered is that the predictions of the ML algorithm should improve (by reducing the error between real and prediction) throughout the evolution of aircraft pairs. In the same way aircraft pairs are getting closer to the Closest Point of Approach (CPA), their prediction should improve due to diminishing uncertainties (aircraft location, weather or wind). The first analysis consists of evaluating how the error differs between the prediction and the real value of MinDis as function of the time to the CPA. Figure 3 shows the evolution of the error for four aircraft pairs. 

It is clear that the errors vary over time. Figure 3 shows that one aircraft performs high deviations without following a specific trend (one error is positive and the next is negative), being the largest deviation from one prediction to the next up to 8 NM. The rest of the samples work pretty well because their error is between ±2 NM and the variations are not important. Although this is not something that should be accepted for a CD that could function as a critical safety tool, it can be solved by smoothing the predictions considering the average value of the last n inputs. 

**Figure 4:**
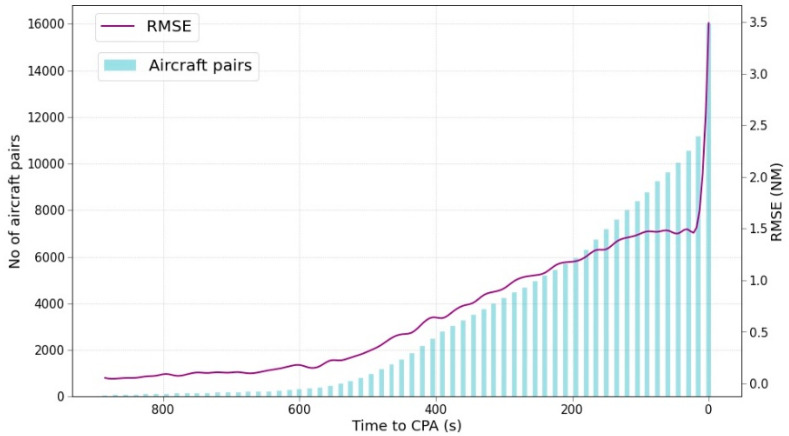
RMSE and aircraft-pair samples evolution.

Neither pair of aircrafts in Figure 3 presents a clear trend that could confirm the previous assumption about the performance of the ML predictor. Therefore, the next analysis consists of evaluating the variation of the RMSE over time. Figure 4 shows the variation of the RMSE throughout the time and the number of samples considered at each timestamp every 15 s. 

Following the previous hypothesis, the evolution of the RMSE can be clearly identified: the closer to the CPA, the greater the RMSE. From 500 s to the CPA the number of samples grows linearly until the last group of samples that almost double the number of samples. The vast majority of samples are located at predictions for 0 s to the CPA. The leap with the previous set corresponds to the aircraft pairs that enter into the airspace and they are diverging, i.e., its initial point is their CPA, or they are flying at different FL. Further work should consider whether these samples should be removed by performing a pre-filtering and the algorithm should focus only on the aircraft pairs that converge in the airspace. Moreover, there is a huge difference between the number of samples from the very far CPA to the last instant that should be more balanced. 

**Figure 5:**
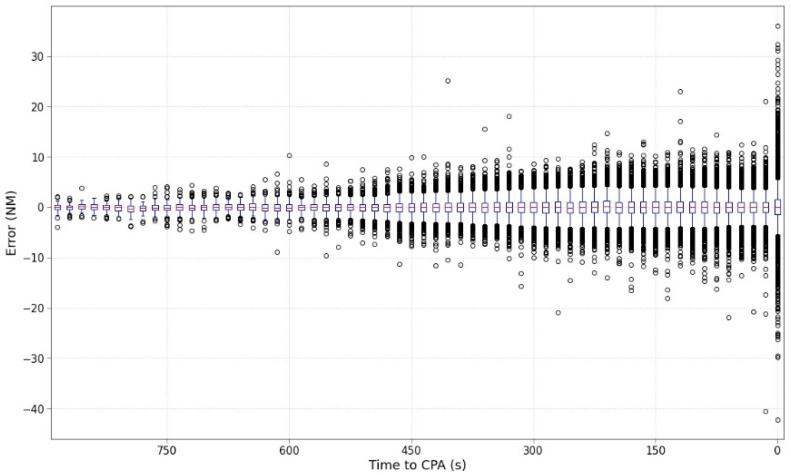
Boxplot evolution of the error every 15 s.

**Figure 6:**
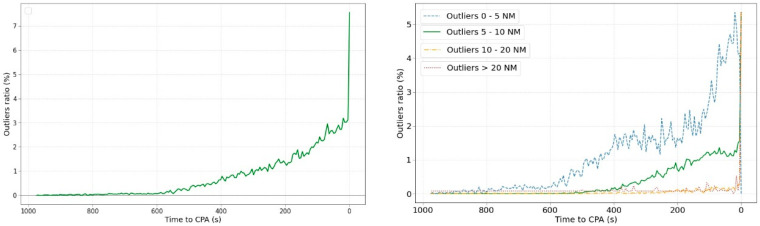
Distribution of outlier’s ratio: (**left**) altogether evolution and (**right**) depending on the stretch.

Figure 5 shows the statistical information (based on boxplots) split every 15 s to facilitate the representation of the results. The mean value is located throughout time very close to the 0-value error, this means that the algorithm works correctly on average. However, it can be clearly identified how the size of the boxplots enlarges accordingly with the number of outliers as the CPA approaches. Figure 6 shows information related to outliers in the predictions. Outliers are data points that are statistically noticeably different from the rest. Herein, it has been detected based on boxplot techniques.

Similar to previous graphics, the distribution of outliers increases in the last times to the CPA. It is notable that the increase in the outlier ratio in the last prediction increases above 7% of the outliers. Analysing in detail the distribution of outliers as a function of their value, the right figure shows their distribution considering four stretches. The densest stretch is represented by outliers from 0 to 5 NM, which represents the best situation. The number of outliers larger than 10 NM is very rare with values lower than 1%. All systems present some quantity of outliers and could be treated by stability processing of the signal. However, an outlier ratio greater than 1% should not be acceptable, although it depends on where the outliers are occurring. Outliers cannot be allowed for separation infringements but could be affordable if they occur for No SI situations, i.e., in cases where the MinDis predicted is 30 NM but the real could be 50 NM. 

**Figure 7:**
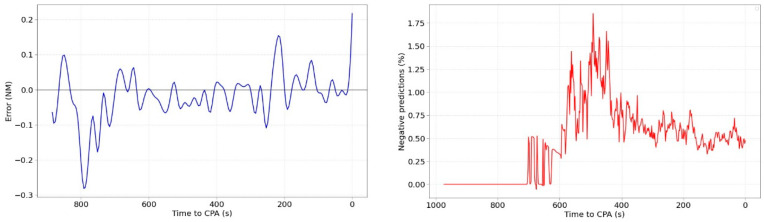
(**left**) Mean values of the prediction error and (**right**) percentage of negative predictions.

Observing Figure 7 left, it can be observed that most of the mean values throughout the evolution are below 0 (negative values). This means that the ML predictor tends to provide a lower value than the real one, that is, the algorithm tends to be conservative. Although this result could be beneficial for safety, it could be on its way around because the number of false alerts could increase. The ATCO would be notified, on average, more times than it should be. Figure 7 right shows the percentage of predictions that provided a negative value, i.e., the MinDis prediction acquires a value < 0. This is an error of the ML algorithm because it has not been trained on negative values (no negative values were in the training data). Regrettably, there are no mechanisms to directly avoid this type of results during the training phase of the algorithm. 

**Figure 8:**
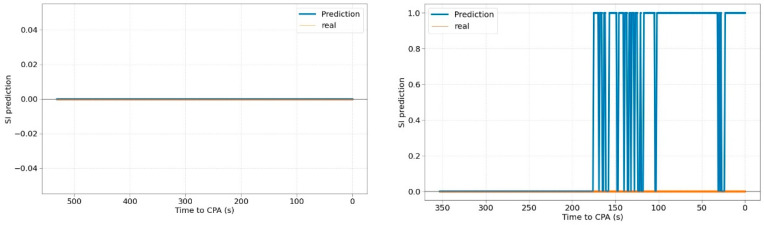
Examples of classification evolution.

Regarding the classification predictions, they can be correct (SI or no SI prediction matches real value of SI or no SI), missed alerts (prediction do not alert of SI while in reality it is) and false alerts (predictions alert of SI while in reality it is not). In this study, missed and false are considered equally important, as they are considered as failures. Figure 8 shows the evolution of the classification results for different pairs of aircrafts. The results of the SI prediction can be 0 (No SI) or 1 (SI).

The left figure shows a stable prediction (No SI) that does not change throughout the time. The figure on the right shows an unstable prediction that jumps from 0 to 1 (SI or no SI). These changes in the prediction occur when the probability of it is very low (around 50%). The stability of the classification predictions has been studied throughout the time. The percentage of aircraft pairs for which the classification predictions are unstable (does not provide the same prediction throughout its evolution) is 20.6%. This value should be evaluated in an in-depth study in further work trying to reduce it or, at least, the number of leaps throughout the time.

**Figure 9:**
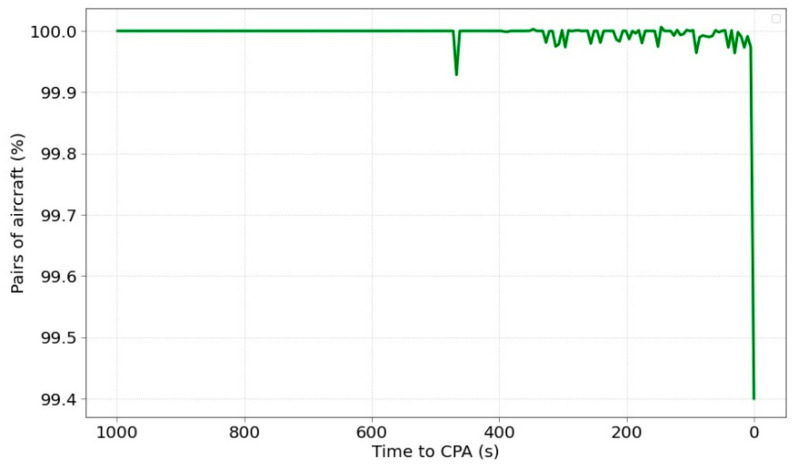
Percentage of SI prediction success depending on time to CPA.

Lastly, Figure 9 shows the evolution of SI prediction success as a function of time to CPA. The performance is rather steady throughout the time except for the last instant that drops the classification rate. This behaviour is fairly similar to the RMSE performance although the error of the SI classification is extremely reduced. 

Therefore, a thorough analysis has been performed to understand the reasons why the ML algorithm degrades or improves its performance over time. There is no clear answer for that, and further work should focus on this area. One of the main limitations is about the last instant the ML make the predictions that should be evaluated in-depth. It is clear that the introduction of time-dependent analysis to AI-based systems is paramount to understand how it works and if the metrics used match the requirements. 

## 5. Conclusions

This work deals with the learning assurance process for AI-based systems in the field of air traffic control. A conflict detection tool has been evaluated to identify separation infringements among aircraft pairs. The CD tool uses ML algorithms to perform predictions based on two approaches: regression techniques to estimate the minimum distance expected and classification techniques to define an aircraft pair as situation of interest. The ML algorithms used to train the model were extreme gradient boosting for classification and regression techniques. The learning process has been validated by following ML approaches and, in particular, the W-shaped methodology provided by EASA. Then, the goal of the work was to dig into the ML algorithm and predictions to understand its performance and to avoid the black-box effect. This implies that, although this work evaluated the results from extreme gradient boosting algorithms, the process evaluated and the results obtained are valid independently of the ML algorithm.

The evaluation performed several analyses considering the number of samples, the rate of error of the predictions and the tendency of the ML algorithm. The initial assumption was that the prediction should improve as the aircraft pairs were getting closer to the CPA. Regression and classification predictions for each aircraft pairs varies as a function of time. Nonetheless, results confirmed that both classification algorithm and regression algorithm performed the other way, i.e., predictions got worse as aircraft were closer. 

Evaluating the validity and adaptability of EASA W-shaped methodology for ML-based systems, the results identified the lack in time-dependent analysis. This is one of the most meaningful results because it was not specified by the W-shaped methodology. This implies that, although EAS developed a good start point for further certification process, it demands to include new analyses. Hence, AI-based systems that depend on the prediction time, RMSE or accuracy cannot be just one number because it varies over time. There is also a relation between the number of samples and the deterioration of metrics that should be studied because it could be related with the time-dependent imbalance of the training dataset. 

Finally, major efforts must focus on the development of regulation and certification of AI-based systems, particularly in aviation. Data analysis is a crucial area for AI-based systems that demand new guidelines and framework to ensure valid performance. 

## Figures and Tables

**Table 1:** Statistical data extracted from the whole database.

	Altitude	∆Altitude	Groundspeed	∆Groundspeed	Vertical Rate	∆Vertical Rate
**count**	2,841,803	2,841,803	2,841,803	2,841,803	2,841,803	2,841,803
**mean**	38,176	1683.21	448.76	622.41	26.09	138.77
**std**	1681.82	1750.47	32.35	275.99	208.71	282.77
**min**	36,025	0.00	343	0.00	−2928	0.00
**25%**	37,000	975	420	466.31	0.00	0.00
**50%**	38,000	1000	445	709.41	0.00	64
**75%**	38,975	2025	475	860.11	0.00	128
**max**	44,950	8530	555	1022.01	2136	3264

**Table 2:** Results from the regression and classification ML models.

	RMSE (NM)	Accuracy	Recall	Precision	F1
**Mean**	3.3	0.99	0.99	0.99	0.99
**Std**	0.3	0.001	0.001	0.002	0.002

## Data Availability

The data can be available on request.
